# The importance of informational, clinical and personal support in patient experience with total knee replacement: a qualitative investigation

**DOI:** 10.1186/s12891-017-1474-8

**Published:** 2017-03-24

**Authors:** Laurie J. Goldsmith, Nitya Suryaprakash, Ellen Randall, Jessica Shum, Valerie MacDonald, Richard Sawatzky, Samar Hejazi, Jennifer C. Davis, Patrick McAllister, Stirling Bryan

**Affiliations:** 1 Faculty of Health Sciences, Simon Fraser University, Blusson Hall 10506, 8888 University Drive, Burnaby, BC V5A 1S6 Canada; 2 Centre for Clinical Epidemiology and Evaluation, Vancouver Coastal Health Research Institute, 7th floor, 828 West 10th Avenue, Vancouver, BC V5Z 1M9 Canada; 3 School of Population and Public Health, University of British Columbia, 2206 East Mall, Vancouver, BC V6T 1Z3 Canada; 4 Department of Experimental Medicine, Faculty of Medicine, University of British Columbia, 10th Floor, 2775 Laurel Street, Vancouver, BC V5Z 1M9 Canada; 5 Burnaby Hospital & Surgical Network, Fraser Health, 3935 Kincaid Street, Burnaby, BC V5K 2X6 Canada; 6 School of Nursing, Trinity Western University, 7600 Glover Road, Langley, BC V2Y 1Y1 Canada; 7 Centre for Health Evaluation and Outcome Sciences, Providence Health Care Research Institute, St. Paul’s Hospital, 588-1081 Burrard Street, Vancouver, BC V6Z 1Y6 Canada; 8 Department of Evaluation and Research Service, Fraser Health, Suite 400, Central City Tower, 13450 102 Avenue, Surrey, BC V3T 0H1 Canada; 9 Department of Physical Therapy, Faculty of Medicine, University of British Columbia, 212 Friedman Building, 2177 Wesbrook Mall, Vancouver, BC V6T 1Z3 Canada; 10 Aging, Mobility, and Cognitive Neurosciences Lab, University of British Columbia, Djavad Mowafaghian Centre for Brain Health, 2215 Wesbrook Mall, Vancouver, BC V6T 1Z3 Canada; 11 Rebalance MD, 104-3551 Blanshard Street, Victoria, BC V8Z 0B9 Canada

**Keywords:** Total knee arthroplasty, Qualitative research, Patient experience, Patient-centred care, Support

## Abstract

**Background:**

Total knee arthroplasty (TKA) is the most frequently performed joint replacement surgery in North America. Patient perspectives on TKA have been investigated in various ways, including finding as many as 20% of TKA patients are dissatisfied with their surgical outcomes. Understanding the patient experience with TKA broadly and in relation to patient satisfaction is a key gap in existing literature.

**Methods:**

We report on the qualitative component of a mixed methods prospective cohort study examining patient experience and satisfaction post-TKA for adults in British Columbia, Canada. Data collection consisted of 45 in-depth interviews about individuals’ knee surgery experiences conducted eight months after surgery. Analysis consisted of thematic coding by multiple coders.

**Results:**

Participants’ descriptions of their TKA experiences were primarily concerned with support, or the provision of aid and assistance. Support was insufficient when their expectations of support were not met; unmet support expectations led to an overall negative TKA experience. Support operated in three key domains: (1) informational support, (2) clinical support, and (3) personal support. Key sources of informational and clinical support included pre-optimisation clinics, surgeons, and physiotherapists. Key topics for informational support included pain, pain management, and recovery trajectories. Personal support was provided by family, friends, other TKA patients, employers, and themselves.

**Conclusions:**

Patient needs and expectations for support are shaped both before and after TKA surgery. Patients with an overall positive TKA experience had improvement in their knee pain, stiffness or functioning post-TKA, had their major expectations and needs for support met during their TKA recovery, and believed that any significant future expectations or needs for ongoing support would be adequately met. In contrast, patients with an overall negative TKA experience had at least one major expectation or need for support not met during their TKA recovery, even in cases where they had good TKA outcomes. Suggested interventions to improve the experience of persons receiving TKA include an expanded patient navigator model, revised pre-surgery educational materials, particularly around pain expectations and management, and comprehensive sharing of other patients’ TKA experience.

## Background

Total knee arthroplasty (TKA) is the most frequently performed joint replacement surgery in North America, with age and sex-standardized rates of TKA increasing over time [1, 2]. Various aspects of TKA have been studied to improve clinical outcomes and reduce costs, including reducing surgical wait times and hospital length of stay [3–5]. More recently, researchers have investigated patient perspectives on TKA, reflecting the current interest in patient-centred care [6–8]. Paying attention to the patient perspective—in this case, focusing on improving the patient experience of care—is also a key aspect of the Institute for Healthcare Improvement’s Triple Aim framework. This framework explicitly states that it is possible to design health care interventions that improve the patient experience while also simultaneously reducing per capita health care costs and improving the health of populations [9]. In other words, improving the patient experience does not have to come at the expense of, and can even augment, other cost and quality goals.

Research on the patient perspective on TKA employs two major approaches. The first major approach quantitatively evaluates patient satisfaction after TKA, finding that as many as 20% of TKA patients are dissatisfied with their surgical outcomes [10, 11]. Factors found to influence patient satisfaction include knee pain, stiffness, and functioning before and after TKA, postoperative complications, and patient characteristics including expectations, social support, age, gender, and ethnicity [10–15]. The second major approach qualitatively investigates particular aspects of the patient experience before and after TKA surgery, including deciding to have or not have surgery [16, 17], waiting for surgery [18, 19], pre-surgery pain [20], pre-surgery education [21, 22], post-surgery pain [23], the hospital experience [24], rehabilitative practices [25], managing recovery [26, 27], and returning to physical activity [28, 29]. While it is helpful that these two approaches to patient perspective research exist, it is difficult to integrate and more deeply understand their results. The existing quantitative work on patient satisfaction does not usually take patient experience into account and the existing qualitative work on patient experience does not usually take patient satisfaction into account. The qualitative work on patient experience also tends to focus on specific aspects of the TKA experience rather than examining the patient experience broadly. New qualitative and mixed methods research can build from this knowledge base through allowing for a fuller account of the patient experience and investigating both patient satisfaction and patient experience without fully constraining either focus by preconceived variables and topics. Including a qualitative approach can also provide rich data on patient meanings and preferences [30, 31] and help strengthen decision-making around system resource use and design [32].

We conducted a multiphase mixed-methods study [33] to improve our understanding of patient experience and patient satisfaction following TKA surgery. This paper reports on the foundational qualitative work from our mixed-methods study investigating patient experience and satisfaction with TKA. Our qualitative investigation asked patients to reflect on their TKA experience broadly, across a variety of aspects of their knee replacement experience, and in relation to their self-reported satisfaction after TKA surgery.

## Methods

The qualitative work reported here is embedded within a mixed-methods, prospective cohort study investigating patient experience and satisfaction with TKA. We recruited 515 adults aged 19 years or older with a primary or secondary diagnosis of osteoarthritis scheduled to undergo primary TKA in British Columbia between April 2012 and August 2013. Study participants were recruited from the mandatory pre-surgical total joint replacement education sessions at six sites across the province, including at least one site in each of the five geographic health regions. Ethics approval was obtained from relevant universities and health regions. For the quantitative component of the study (not reported here), each participant completed a pre-surgery, paper-based, self-administered, English-language, questionnaire and most completed additional surveys at 6 and 12 months post-surgery (91 and 88% of baseline, respectively). Questionnaires included demographics and patient-reported outcome measures about pre- and post-surgery pain, stiffness and functioning, health status, expectations, and satisfaction. For the qualitative portion of the overall study, we conducted 70 semi-structured, in-depth interviews with 57 purposefully selected individuals either once or twice at 8 and 14 months post surgery (*n =* 45 persons interviewed at 8 months, *n =* 25 persons interviewed at 14 months, with 12 of the 25 persons interviewed for the first time at 14 months). The number of possible interviews overall and at each timepoint was established prior to collecting data due to resource planning constraints. We anticipated this sample size, which included 8–10 participants per health region (both urban and rural), would be necessary for reaching informational redundancy on key themes and key TKA outcomes and experiences, and for achieving maximum patient variation [34, 35].

This paper focuses on the 45 individuals interviewed about their TKA experience 8 months post-surgery. Our qualitative data are rich and multi-faceted; restricting our initial analytic focus to first-time interviews at 8 months post-surgery allowed us to deeply understand major issues in patients’ initial post TKA-experience. Additional analysis of the qualitative and mixed methods data will build off of the foundation established in this paper—including understanding the effects of time on patients’ post-TKA experience and satisfaction—and will be reported elsewhere.

### Sample selection and data collection

To create the qualitative sample from the quantitative cohort, we considered all cohort participants for inclusion other than 6-month survey non-respondents and those having survey completion assistance. We interviewed in every provincial region multiple times and interviewed as many persons as possible who reported dissatisfaction with their TKA results on their 6-monthpost-surgery questionnaire. We further purposefully sampled for maximum variation on other key characteristics from our survey data and associated literature, including sex, ethnicity, employment status, self-rated health, and various pain, functioning, and emotional health measures. Those purposefully selected were approached by letter followed by telephone call to schedule an interview. Interviews took place where convenient for the participant, including participants’ homes and medical clinics. Interviews were conducted in English by experienced interviewers. The semi-structured interview guide was designed to understand the individual’s knee surgery experience and outcomes. Interviewee-specific probes were also created based on their answers to the baseline and 6-month surveys. Interviews generally lasted 45–65 min. Participants received an honorarium at interview completion. After leaving the participant, the interviewer completed a standardized interview debrief on the key information learned and suggested interview guide revisions. The interviews and debriefs were digitally recorded and transcribed.

### Data analysis

All transcripts and debriefs were thematically coded using NVivo software (NVivo qualitative data analysis software; QSR International Pty Ltd. Version 10, 2012). The coding scheme was initially developed through two coders (NS, ER) independently coding the same transcripts and debriefs and constructing a thematic coding framework through consensus and input from a third coder (LJG). Remaining transcripts and debriefs were coded by one of four coders (LJG, NS, ER, JS) with new codes created when needed to reflect new concepts. Coders met on a regular basis to discuss analysis with key decisions. Text coded at key codes was also regularly reviewed and discussed by other team members to further develop the analysis and provide a check on coding consistency and construct validity. Once all the transcripts were coded with the initial coding scheme, combinations of up to five team members (LJG, NS, ER, JS, SH) met on multiple occasions to discuss key themes and their relationships with the goal of arriving at a higher abstraction of key themes. Individual coders then reviewed text coded at key codes to further develop relationships between key themes and identify representative quotations with results further discussed in larger team meetings. Reflective memos were constructed throughout. Our analysis approach meant that key codes and significant portions of interview transcripts were reviewed in multiple ways by multiple team members. This multi-step coding process helped to ensure the rigor of our analysis [34, 36].^1^

## Results

We purposefully sampled 65 participants from the overall cohort to obtain the 45 persons interviewed approximately 8 months after their TKA surgery. Thirteen persons declined participation due to worsening health or other reasons and 7 were not contactable. Post-surgery interview timing varied based on scheduling, averaging 7.9 months (minimum 6.6, maximum 9.4 months). Each health region was represented by an average of 9 persons (minimum 7, maximum 11). Demographics and other key details of the 45 participants are provided in Table 1. Given our focus on understanding patient dissatisfaction, we oversampled from the 15% of those who reported TKA dissatisfaction in their 6-month survey, resulting in 58% of the 45 qualitative interviewees having reported dissatisfaction (i.e., reporting neutral, dissatisfied, or very dissatisfied on the 5-point dissatisfaction scale). We secondarily oversampled those who indicated that they were uncertain or would not be willing to have their TKA surgery again if they could go back in time (44% of the 45 qualitative interviewees vs. 12% of the quantitative cohort). Our qualitative sample otherwise roughly mirrors key distributions in the overall cohort, with over half of the sample being women, having married or common-law status, a household income below $60,000, or North American or European ethnicity. Over half of both our qualitative and cohort sample also were experiencing their first knee surgery or had waited more than 12 months from the onset of their knee symptoms and first seeing their TKA surgeon.

**Table 1:** Participants’ Descriptive Details (*n =* 45)

		Count
Age, average	65 years	
Sex	Female	30
	Male	15
Marital status	Married or common-law	34
	Widowed	3
	Single, divorced or separated	8
Household income	< $40,000	12
	$40,000 to < $60,000	10
	$60,000 to < $80,000	10
	$80,000 or more	10
	Missing	3
Education	High school or less	19
	College/technical school	12
	Undergraduate degree	3
	Graduate degree	8
	Other	2
	Missing	1
Ethnicity	North American	33
	European	8
	South Asian	2
	Central/South American	1
	Other	1
Received TKA surgery on first or second knee	First knee	32
	Second knee	12
	Missing	1
Time between knee symptoms and first time seeing surgeon	<6 months	9
	≥6 to < 12 months	9
	≥12 months	25
	Do not remember	2
Satisfaction with TKA surgery results, self-evaluated 6 months post-surgery^a^	Very satisfied	8
	Satisfied	10
	Neutral	15
	Dissatisfied	9
	Very dissatisfied	2
	Missing	1
Willing to have TKA surgery again, self-evaluated 6 months post-surgery	Yes	25
	Uncertain	12
	No	8

^a^The 5-point satisfaction scale can also be reduced to a 2-point satisfaction scale where satisfied is composed of those who answered “very satisfied” or “satisfied” on the 5-point scale and dissatisfied is composed of those who answered “neutral,” “dissatisfied” or “very dissatisfied” on the 5-point scale

**Fig. 1:**
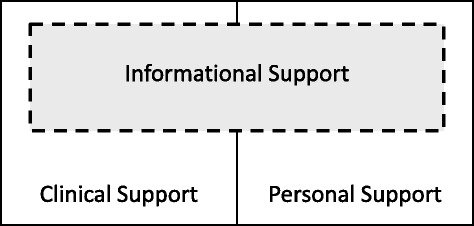
Key domains of support in patient experience of total knee arthroplasty

Participants’ descriptions and sense-making of their TKA experiences were primarily concerned with the provision of aid and assistance, a concept we label “support.” Support was deemed to be insufficient when their expectations of support were not met; unmet support expectations often led to an overall negative TKA experience. Support expectations were both formed in advance of surgery and in response to emergent needs after surgery. TKA patients’ experiences in this study primarily operated in three key domains: (1) informational support, (2) clinical support, and (3) personal support. These domains interact with each other (Fig. 1) and a deficiency in one domain can sometimes be compensated for by another domain, as explained below and through illustrative quotations from varied participants (Tables 2, 3 and 4).

**Table 2:** Informational support illustrative quotes

(1) Pre-surgical education session as a source of informational support
“The [education session] is really informative….They prepare you for everything. If I went to the hospital without this program and woke up with my leg looking and feeling the way it did, I think I might have wanted a new limb.”
“I went to all the pre-surgery meetings…But nobody ever really said, ‘This is not a real knee. This is not going to be the same as your other knee was. There will be limitations.’… I did read all the literature but nowhere did I see that said.”
“The [education session] was pretty good but not good enough. I don’t think we really got enough warning about how much assistance you need afterwards…But maybe they can’t tell you what to expect because there’s so many differences in people too—it would take forever and maybe scare some people needlessly.”
“I still don’t think that they explained how painful exercises are going to be at the pre-op session. You are told, but it doesn’t sink in. I think that should really be pushed. It is going to be painful but you can’t do any damage. Like, once the knee is in place, you can’t really harm it.”
(2) Surgeons as a source of informational support
“The one you really want to rely on is of course the surgeon. [You want to ask] ‘What did you do to me?’ or ‘What are you going to do to me?’“
“They are always in a hurry,… [My surgeon] showed me the x-ray. ‘And this is fine. It’s fine. You’re going to be fine.’…You are just a number and you just go and it’s quick, quick, quick.”
“I think the hardest thing was [my surgeon]’s so hard to talk to…I think that probably was a lot of my problem, not feeling like I was given enough information.”
“I only saw [my surgeon] a few times…a very nice person, very friendly to me. I wish I could see him more to get more information…but they have so many patients, [the visit is] so very fast.”
“The surgeon, when I first met him, I thought, ‘Boy, that guy’s got no personality.’ When I got to know him I realized he does, but he’s a busy man. They are very busy. He’s willing to answer any questions but if you don’t have the questions to ask, how can they answer them?”
“I wanted to know why I was so numb in my knee and [the surgeon] didn’t answer me. He just said ‘You’re going to have to give it time.’“
“You have to be really prepared and aware what you want to ask. You got to go in prepared because you get a little nervous. You get intimidated by these guys.”
“[My surgeon] walked us step by step. He showed me what the surgery would do and what it would look like and then he showed me the x-rays of my knee and he explained everything that was going on…I can’t praise him enough.”
(3) Other health care providers as a source of informational support
“My family doctor is fine but he—perhaps because of the little bit more complexities in this case, he really didn’t have any opinions of his own about things. He really deferred everything to the surgeon.”
“When I went for physio, the therapist kept on saying, ‘It’s going to be a year.’ And so that gave me hope too because when I first went there, I thought I’d be better already. I would have thought ‘A month has gone. What’s wrong here?’“
“The [physiotherapists] here tell you to get on with the exercises and don’t back off on that. They did point out quite emphatically that if you have pain, use the medication. Don’t back off on the exercise because of pain. If the knee hurts, take a pill. Don’t stop bending it.”
“The physiotherapists set you up with a program. You’re only allowed to go for three visits. So you are cramming in three visits all these exercises which you are supposed to do. And rather than following up, people just go back to their old patterns because no one is checking up.”
(4) Other TKA patients as a source of informational support
“We are all comparing scars [saying] ‘Oh, your scar is so much nicer than mine.’”
“If I had met [another TKA patient] who would have told me the honest truth—‘This could happen’ or ‘I had this happen’ or ‘There’s quite a bit of pain at first,’ you know, this sort of thing. I might have had more questions to ask [the surgeon].”
“My girlfriend is getting it done so she was asking me different things… I did tell her to go to all the physio…. I said that through other people that were in physio, I did hear that [her surgeon] was a good doctor.”
“I think seeing where other people are at [physiotherapy] gives you incentive too and makes you say, ‘I should be able to do this.’ Or ‘I should be working at it harder.’”
(5) Informational support for pain expectations
“I don’t think it’s stressed enough and I don’t think I ever read or heard before I had the surgery that the pain is not going to go away for a year. I thought [it would go away] in a month”
“I thought it would be better than it is…The twisting pain I’m hoping will get less but it’s still pretty severe…I thought my knee wouldn’t hurt when I walked down the stairs, and maybe it won’t, given some more time. I am constantly told ‘Wait, wait, wait.’ so, I’m waiting. I just expected less discomfort after this period of time.”
“The meeting at the hospital before you went in for surgery where they were explaining kind of what is going to happen. And they kept saying, ‘Oh, yes, you’ll have a little pain.’ I wish they had been a little bit more honest as to *the amount of pain*.”
“I got mixed messages particularly when I went to physio. One person would say to me ‘Oh well, don’t do it if it hurts.’ Another person would say ‘Well, that’s the way it is.’…It’s a bit confusing.”
“[After my first knee replacement] I was afraid to push it too hard because I didn’t know if I was going to do damage because of the pain. This time [for my second knee replacement] I knew I couldn’t really do any harm…I think probably for a lot of people the pain with the exercises, they are not prepared for it.”
(6) Informational support for pain management
“I’m frustrated [by the pain]… I’ve been back to my GP a few times saying, ‘Come on, there’s got to be something.’ ‘No, you are doing great.’ I go to physiotherapy. He says, ‘Oh, look at the movement in your leg. You are doing terrific.’ Okay, I am doing terrific but it hurts.”
“The physiotherapist said it’s breaking down scar tissue which tends to form. You have to break it down to get the range of motion. And that gets uncomfortable. So bear with it. Use the pain killers as necessary but don’t let pain restrict your recovery.”
“I kept on talking to [my family] doctor saying, ‘I don’t want to get [addicted].’ They say, ‘Take the pain medication, the pain medication. Manage it so you can move it.’ And I said, ‘Well, I don’t want to get addicted to it.’ ‘Oh, don’t worry about that, don’t worry about that.’ But I did worry about it.”
“I think that if I’d had somebody I could call, even a couple of times like now and say, okay, it’s seven months, I’m in pain, the swelling is really bad today, what the hell do I do?”
“There is nobody to talk to. You call the surgeon and unless there is like a major problem they don’t want to hear it from you because all they care about is what the x-ray shows and the x-ray shows perfect. It’s fine. The GPs, they didn’t do the surgery so it’s more pain control—like, ‘Do you want stronger pain pills?’ And I said no. I don’t want to just cover up the symptoms. I need to know what is going on. So you can get on the internet and check things, but there is nobody to really talk to about the pain, the swelling.”
(7) Informational support about recovery trajectories
“Unless I’m the exception. I don’t know if everybody has [these problems]. ….It would be nice for them to say, ‘Okay. This is the scenario. Some people may get full movement back but some people may not,’ you know. If they could let you know those options but they didn’t.
“My brother had both of his [knees] done two years previous and a friend of mine had hers done and the neighbour across the street had hers done with the same doctor that I got it done with so I kind of knew what to expect.”
“I should have sat down with [my neighbour] longer because he’s had his knees done…I’d like to have a phone-a-buddy, to phone somebody that’s had an knee operation the same time I did and ask ‘How’s your recovery going?’“
(8) Informational support about other post-surgery issues
“Nobody said anything about the clicking…It kind of [worried me] because I was wondering if there was something wrong, that it shouldn’t be like that. And, of course, I got told it was quite normal. Everybody’s knee pretty much does it. And he explained why, which was good. Once I got the information and I understood that it wasn’t a big deal, it was fine.”
“[The surgeon should] take some time to really explain to somebody what’s really going to happen, like, what your expectations should be. You may not have the pain you had before but this knee is going to make noise when you walk. This knee is going to feel like it’s crunching inside your leg. You’re going to have quite an ugly scar. You’re knee won’t be shaped the same as your other knee anymore.”
“This [part of my knee] is still numb. I asked about that. [My surgeon] said, ‘Oh, it may never come back.’ It looks very different than my other knee. I know I’ve got ugly knees but it’s small. This is smaller. It gets warm still and that’s something the physio said is not good… [My knee is] a lot better than it was but it’s certainly not as good as I would like it to be.”

Italics indicate word was emphasized by participant

**Table 3:** Clinical support illustrative quotes

(1) Surgeons as a source of clinical support
“[My surgeon] has a good reputation. The hospital thinks the world of him…So I went to visit him and it was just a great match. He covers all the bases and tells you everything. There’s no secrets, no big surprises. He said if everything goes well, I’d only be in the hospital three days. That’s what it was.”
“[My surgeon was] not really reassuring or anything. Very matter of fact…very ‘It’ll be this way. If that doesn’t happen, this will happen and we’ll do it that way.’ And basically that was it…he wasn’t very personable…It wasn’t any kind of conversation. It was very quick.”
“[The surgeon] said, ‘I just wish I had more patients that were like you, that were healing quicker.’…I felt good because he did a good job and I felt good because I’d done a good job doing my exercises and everything. It was a win for both of us.”
“[My surgeon] is so caring…even when I am crying he is like, ‘Oh, we’ll do this. We’ll get through it.’…I knew that he was going to help me.”
“I think the minute he heard or saw my psychiatric file, I think he probably thought this person isn’t worthy of a knee replacement.”
“The surgeon is useless to get information out of…6 weeks [after the surgery] I went in there and he said to me something which I didn’t understand and I’m sorry I never pursued it.”
“I’d saved up these questions. I wanted to know if it was cement or screwed in. I wanted to know if the scar was the way it should be, if the numbness should be there. He answered my questions but in a very different way than I would have assumed he’d answer them. I would think that rather than make it sound so ordinary—this isn’t an ordinary thing.”
(2) Physiotherapists and physiotherapy as a source of clinical support
“Physiotherapy is the one thing you can count on.”
“I went to physio twice a week and each week I could tell, getting in and out of the vehicle and walking into the hospital, I could feel that it was getting stronger.”
“[The physiotherapist’s] attitude was all help. If you needed help, she helped. Very positive, saying things like ‘Work through the pain, you’re doing great, just push a little harder.’”
“I went to rehab at the hospital and I could have done it as long as I wanted. They were fantastic. And it was all covered. I never got asked to pay for anything, it was all covered.”
“One of the things they worked on [in physiotherapy] that I found very helpful, so did other people, was they developed a camaraderie, this big family get-together type of thing, to talk to people, compare notes and get a little encouragement from patient to patient. So it wasn’t just an isolated one-on-one therapist to patient. There was a lot of dialogue between patients.”
“They were sending in a referral to [hospital name] for physio. I was given a phone number to call. So the first week home I called and they said there is nothing available yet…I was getting very desperate and in about the fourth week I started calling other hospitals… I was almost in tears. I was at my wits end, didn’t know what to do. And the woman in reception there said, ‘This isn’t acceptable, I’m going to talk to the physio and I’m going to call you back. I’m sure we will fit you in.’ She phoned back the next day…Without them—if they would have taken the same attitude as everybody else—I probably still wouldn’t be able to bend my knee.”
(3) Family doctors as a source of clinical support
“I have a GP who has been with me through the whole pain medication process, to the reduction of opiates, to the pre-surgery consult through the referral. He took my staples out at the end of the surgery. He followed me along through the recovery process post-surgery to make sure there was no infection. He followed along with the physiotherapist’s recommendations.”
“I think my GP does know [that I still have unresolved pain in my knee]. I think he does. He knows I am under a lot of stress, a combination of the pain and being a caregiver. But there is nothing he can do. He can only give me so many Prozac and so many painkillers.”
“I did go to the GP about my knee and asking him for advice on what I can do because the system isn’t doing it. And he suggested Aquafit.”
“I don’t talk to my family doctor about it because he’s not interested. That’s the surgeon’s problem. [My family doctor] doesn’t want to get involved.”

**Table 4:** Personal support illustrative quotes

(1) Family and friends as a source of personal support
“My son has been very helpful. He’d do the shopping or the laundry and cleaning or drive me places to my appointments.”
“My wife and daughter were trying this idea of one on each side and then three people abreast across can’t go up the staircase. But our nurse friend knew how to negotiate all that.”
“There are people here in the co-op. They were only a phone call away if I needed anything. They’d phone, ‘I’m going grocery shopping. Do you need anything?’“
“I needed to get to a physio…I didn’t want to impose on [my friends] to drive me over there and sit for an hour. But I couldn’t really trust my husband because he’s got dementia.”
“The whole surgery thing made [my husband] very anxious. So his daughter had come to stay with us for a couple of days…and once I had the surgery she left. So I had to get back on my feet almost immediately and I was driving within 10 days, you know, could just barely move my foot but I could move it enough, to drive the car.”
“[My friend] said, “Well, we can’t go walking or do anything because you’re an invalid. You can’t walk.’ So my social life has gone downhill.”
“Our church family was so supportive too…It’s incredible, the cards, the phone calls, the meals I would get…it shows they care. I think that is such a huge part of recovery.”
(2) Other TKA patients as a source of personal support
“[My friends who had knee surgery] knew what it was all about and they told me how important it is. ‘Do your exercises. Don’t put them off.’ And when I could see how well they were doing, it encouraged me.”
“I talked to more people that are waiting to have [their knee replacement] done to encourage them. I find that a lot of people are scared and I try to encourage them because I say you just won’t believe how you feel the day after your surgery to have that pain anymore.”
“I spoke to about half a dozen people that had it and they were all walking around, they were fine, they were back to playing sports and doing whatever.”
(3) Self as a source of personal support
“I was prepared and knew you need to have a toilet riser, you need to have a cane, you need to have a walker… I even went to the Red Cross and got everything there.”
“One of the things I learned [before surgery was] change your life before [surgery] and you’ll heal better. Which I did. I stopped drinking. I’m not a big drinker but I totally stopped alcohol.”
“I was very good about doing all my exercises every single day, twice a day as they told me. I went to physio on my own after I ran out of physio at the hospital, which I have no coverage for so that was another three hundred and fifty bucks [out of my pocket.]”
“I was also an active participant in the process which I think has got to be one of the keys to it. You can’t be a passive person and let them kind of do things to you because ultimately you have to be responsible for your own rehabilitation and recovery, be involved in it right from the very beginning.”
(4) Employers as a source of personal support
“The union is supportive. The company is supportive… People try to be as accommodating as they can if somebody needs help.”
“So I postponed going to work for that month, plus I work for a doctor and she knows what is involved, and she said, ‘No, definitely take another month off, take the time that you need.’”
“I am not at full time yet. I am working five hours a day. I hoped to increase that but my knee would keep flaring up and I couldn’t attend at all. Work has agreed to rent a recliner. Sometimes my knee is swollen and I need to keep it up. That’s the difficult part.”
“We have a really good extended health care program. They covered everything….I had to get a pool pass. He just said, ‘Just send us a receipt. We’ll cover you.’”
“I’ve had some issues with my employment, about getting back to work and its very aggravating and its very stressful…after two months they were phoning me, ‘You can come back to work.’… But then I said I also have physio. There’s where they have a fine line: you go to physio and now you’re on sick time again.”
“I went back to work and did full time for three months and I just crashed. I couldn’t do it anymore… My manager was…unsympathetic.”

### Informational support

All participants noted the importance of information about TKA preparation and recovery. Patients received information through formal clinical sources, such as pre-surgical education sessions and health care providers, and informal personal sources, such as friends and family, the internet, and, when applicable, their experience with having already received TKA on their other knee.

Each participant identified the pre-surgical education sessions as a key form of informational support from a clinical source. The provided information was often also described as insufficient—many participants wanted more information than routinely provided at the sessions to be better prepared for TKA recovery and to actively participate in their own care. Some participants further said that their education session information was not meaningful as it was difficult to understand or remember the instructions or it was difficult to reconcile the different messages they received from different presenters. This information was further complicated by having hip-replacement patients at the same education session as knee-replacement patients. Participants also reflected on how they were overwhelmed, anxious, or scared before the surgery, which made it challenging to retain information from the education session.

Surgeons were both expected and actual key clinical sources of information about TKA preparation and recovery, although many participants wanted more information from their surgeon than they received. Multiple patients described their visits to the surgeon as too “matter of fact,” emphasizing checking physical functioning. Participants described surgeons as not readily providing wanted information and not having or making time to answer questions. This situation was exacerbated by patients feeling overwhelmed by their visit to the surgeon, which often led to patients not asking their prepared questions. Participants noted and appreciated when a surgeon took time to provide sufficient and helpful information. A few participants recounted that even though their TKA outcome was not as good as they expected, the time and information provided by their surgeon both before and after the surgery improved their TKA experience. Regardless of the surgeon’s ability to provide information support, patients typically saw their surgeon a few times following their surgery which restricted patients’ ability to use the surgeon as an information source.

Other health care providers could be valuable sources of information post-TKA but were inconsistent sources of information support. Many participants recounted that their family doctor was no longer involved once the decision to have TKA had been made. Some sought advice from their family doctor after their TKA surgery if their surgeon was unavailable but found that their family doctor provided no or limited information. Physiotherapists provided information about post-TKA exercise and recovery and often interacted with participants multiple times after the surgery, but some participants still felt that the exercise information was not comprehensive enough. One hospital had physiotherapists make home visits to TKA patients after their surgery which participants found helpful for understanding their recovery in their home context.

Talking to other TKA patients was another form of informational support. Although a few participants expressed a preference for dealing with things on their own, the majority of participants stressed the importance of talking to other patients with previous TKA experience. Patients shared information about surgeons, types of treatment, exercises, and healing and recovery strategies and trajectories. Such information sharing was sometimes a response to insufficient information from clinical sources.

The most frequent type of informational support identified as needing improvement was information on pain expectations. Many participants expected that the surgery would alleviate their pain and were surprised and unhappy when they experienced intense pain after their surgery, particularly when pain was long-lasting. A few people thought that when the arthritis was “taken out” of their knee with the surgery that their pain would be completely gone. Some participants said that their pre-surgery education session did not tell them they would experience post-TKA pain; others said that while their education session did provide information about pain, not enough information was provided. Participants on their second knee TKA experience illustrated the empowering nature of this knowledge, recounting that they knew this time around to expect significant pain and to exercise through it. Despite their interest in having more information about pain in advance of their surgery, some participants felt that fully forewarning others might stop them from having surgery.

Participants also expressed concern about inadequate information regarding pain management. Pain management education was sometimes offered by physiotherapists during rehabilitation. Some family doctors provided assurance about pain medication addiction concerns and home care nurses provided education about icing techniques. Despite the existence of these forms of assistance, inadequate pain management information support was a frequent issue for participants. When reflecting on how they could have better learned about pain and pain management, many participants suggested that TKA patients should have access to a “go to” clinical person to answer patient questions. The clinical expertise of this person was left unspecified, although many participants also expressed that surgeons should be more available to patients to discuss pain and other recovery concerns.

Multiple participants wanted to understand the variety of TKA recovery trajectories so they could be assured they were on some sort of a track to recovery, even if it was not the ideal track or an ideal recovery. To supplement the inadequate recovery trajectory information, many participants compared themselves to other TKA patients they knew. When their recovery experience was worse than others’ experiences, participants did not know how to make sense of this mismatch and wanted clinical support in understanding their problems. Many participants suggested that a formal patient buddy program would be helpful, where patients could be paired with a former TKA patient to normalize the recovery experience. Some participants had already started doing that for others in an informal way.

Other areas participants identified as needing additional information support included post-surgery issues like knee clicking, infection and scarring; post-surgery exercise and functioning; and alternative and supplementary rehabilitative options.

### Clinical support

Patients expected that surgeons would be key helpers for making sense of their TKA experience but few participants were provided with this clinical support. Many participants wanted more personal and higher quality interactions with surgeons where ideal interaction examples consisted of both emotional support and support with their health needs, including information support. Most patients wanted a surgeon who was both a skilled technician and an empathetic individual, yet participants often described surgeons as mainly providing surgery specific support, with little to no effort at building rapport or making the patient feel like an individual. A lack of personal interaction often impeded patient reassurance and many participants had a hard time understanding why their questions and concerns were unanswered or diminished.

Patient sense-making was further challenged by mismatches between the patient’s and surgeon’s perspectives. The first type of mismatch was where the surgeon lacked empathy for the patient’s experience, including times that patients learned for the first time post-surgery that their knee should have been replaced much sooner than it was and their post-surgery pain indicated a longer recovery timeline. Another type of mismatch was demonstrated when the surgeon did not appear to be seriously investigating the patient’s unresolved post-surgery problems, which often left the patient frustrated and confused. The lack of availability of the surgeon post-surgery was a third mismatch and was sometimes interpreted by the patient as the surgeon not caring about or not believing the patient. Some participants reported that mismatches resulted in losing trust in the surgeon and expecting that future interactions would be as unsatisfying as in the past. Participants sometimes attributed mismatches to the power imbalance between the surgeon and patient, describing the surgeon as condescending or arrogant.

Physiotherapists and physiotherapy services provided key clinical support. Physiotherapy helped patients regain mobility and resume their regular activities. Many participants felt that a “good physiotherapist” was critical to properly recovering from surgery. Good physiotherapists had effective communication skills, treated each patient as a person, had time for patients, and tailored the services to the patient’s needs, including extending the number of sessions provided to the patient. Hospital-based physiotherapists were valuable due to their proximity to the surgeon, although some private-practice physiotherapists were valued for their flexible schedules. Physiotherapy was also a place where patients could share information, interact with others, and benchmark their recovery with other TKA patients.

A few patients received inadequate physiotherapy support, usually resulting from not getting scheduled for physiotherapy until long after their surgery. Most of these examples were from patients having their surgery outside of their local catchment area and then trying to receive outpatient physiotherapy close to home, although a few participants were not scheduled for outpatient physiotherapy even when their surgery was at their local hospital. Some of these unscheduled patients were assisted by persons within the health care system to eventually get physiotherapy, although the patients had to first advocate for themselves to multiple points in the system before finding an advocate in the system.

Although they played a minor role in post-TKA clinical support, family doctors sometimes assisted with pain medication or provided additional recovery advice, such as suggesting other rehabilitative activities like massage therapy and water exercises. Other family doctors were reluctant to provide information; participants perceived this was because the family doctor did not want to get involved in the “surgeon’s business.”

### Personal support

Family and friends were important sources of personal support for a variety of activities. Participants recounted needing much assistance with activities of daily living after TKA surgery. When first sent home from the hospital, participants described needing assistance turning over in bed, bathing, using the bathroom, and using stairs. Participants were initially unable to drive after surgery; some had family or friends drive them to health care appointments while others relied on public transportation. Family and friends also went grocery shopping and prepared meals. Some family members—male spouses/partners in particular––and some friends were not capable of providing personal physical support. Reasons for this lack of capability included: being anxious, feeling unskilled, not understanding what support was needed, their own physical impairment, and being busy in their own lives.

The physical support provided by family and friends often also helped patients feel emotionally supported. Participants also described explicit emotional support provided by family and friends through visiting and going out for meals or social activities. Other TKA patients also provided key emotional support through validating participants’ feelings and providing encouragement about recovery.

Participants further recounted the importance of self support. Many undertook pre-surgery preparation of their home for post-surgery safety and convenience. Many felt highly responsible for their own healing and recovery and expressed this responsibility through creating pre- and post-surgery exercise routines, undertaking lifestyle changes such as dieting and losing weight, maintaining hobbies, and keeping a positive attitude despite post-surgery challenges. Self-support was also expressed through advocating for better treatment or extra attention from providers and specifying from whom they received care.

Employers could be additional sources of personal support for working participants. Many working participants described their employers as supportive and understanding of their situation, including allowing them to work at home. Some participants also had insurance through their employer that covered all or some of their health care expenses, such as extra physiotherapy. Other working participants had negative experiences with their employers, including being unable to take enough sick days for recovery and having to return to work with minimal physical accommodation.

## Discussion

We found that patient experience of TKA can be conceptualized in terms of patient needs for informational, clinical and personal support, where patient expectations for support is shaped both before and after TKA surgery. Patients with an overall positive TKA experience had improvement in their knee pain, stiffness or functioning post-TKA, had their major expectations and needs for support met during their TKA recovery, and believed that any significant future expectations or needs for ongoing support would be adequately met. In contrast, patients with an overall negative TKA experience had at least one major expectation or need for support not met during their TKA recovery, even in cases where they had good TKA outcomes. Patients with overall negative TKA experiences sometimes also believed that any significant future expectations or needs for ongoing support would not be met in an adequate way, usually based on having already experienced multiple instances of inadequate support.

Having appropriate support could help address post-TKA pain, functioning or stiffness problems, which were identified as significant challenges by several participants. In all cases where our study participants reported having post-TKA knee issues, having caring and informative clinical input, especially from the surgeon, made the participant more likely to report positive experiences than would be expected given her or his knee problem. Participants were clearly comfortable with knowing that recovery would take time, that not everything would work out perfectly, and that they had a role to play in having a good TKA experience. In other words, participants were very comfortable with taking on their part to support a positive TKA outcome, even without perfect knowledge of all that would be expected of them. Along with assuming their own responsibility, participants expected health care providers and the health care system to support them to achieve positive TKA outcomes across a variety of patient experiences, including difficult, challenging, or unusual patient experiences. Where health care providers did not support this approach—such as telling the patient that everything is fine with their knee when the patient thinks their knee is far from fine—our study participants challenged this thinking, suggesting that clinicians and the system needed to expand the boundary of when they end their support for the patient.

Participants in our study were clearly asking for clinicians and the health care system to adopt a more patient-centred approach across their surgery and recovery experience. Improving patient-centredness is the responsibility of all involved in the health care system [6, 8, 37]; surgeons are neither the sole problem nor the sole solution, even though surgeons played a key role in participants’ narratives. The current system structure positions the surgeon as one of few resources the TKA patient can turn to for problem-solving, which carries with it all of the limitations and vulnerabilities of single node systems, including bottlenecks [38]. This does not need to be the system structure of the future. Orthopedic clinics and hospitals could offer additional options for patient problem-solving. One intervention that could fill this gap would be an expanded patient navigator for TKA patients, where the patient navigator role is held by a clinician such as a nurse practitioner who can provide patient care and education and liaison with surgeons, physiotherapists, primary care, home care, and social services. Other possible gap-filling interventions could expand the offered support beyond the health care system, such as implementing the “TKA buddy” program advocated by participants in our study, where current TKA patients are paired up with another current or previous TKA patient to provide a venue for informational and social support. Our study participants also suggested that pre-surgery educational materials could be improved, including sharing the recovery stories of multiple TKA patients to illustrate the variety of TKA patient trajectories and provide another form of informational support, particularly around benchmarking and normalizing individual situations and experiences.

Our results resemble other qualitative studies showing that a mismatch between patient expectations and TKA outcomes are associated with negative patient experiences and patient dissatisfaction with knee replacement [24, 26, 27, 39–42]. One quantitative review also came to the same conclusion [14] but a second quantitative review found that no such relationship after adjustment for confounders [43]. Although these two quantitative reviews used a different set of studies, we suggest their different conclusions result more from the inadequate conceptualization of patient expectations, particularly around post-surgery care. Our study showed that patient expectations around TKA outcomes were not always related to the knee itself. Patient expectations, particularly post-surgery, were also related to receiving clinical support for resolving knee problems. Participants in our study who reported problems with their knee and yet were told by their surgeon that their knee was fine did not have their expectations of clinical support met. Similarly, managing patient expectations is in part about providing appropriate and sufficient information to the patient throughout the TKA process [44]. Our study participants’ comments about how more advance knowledge about pain expectations and management would have more positively shaped their experience is one example where information support can lead to good management of patient expectations. Information support can also lead to shared decision-making, or active participation by both patients and clinicians in care decisions, which has a variety of positive outcomes, including improved patient experience, satisfaction, and other outcomes [22, 45, 46].

Our results also resemble other qualitative studies in documenting and specifying the importance of a variety of types of support, although our study differs from previous literature by enhancing, elaborating and unifying a variety of expressions of support as conceptualized in other patient experience studies in joint replacement and osteoarthritis. For instance, Westby and Bachman’s investigation of rehabilitation practices and outcomes from the perspectives of hip and knee replacement patients and providers found that “[rehabilitation] takes all kinds of support.” Although they did not specify categories of support within the “all kinds” description, they did provide examples that directly map onto our study’s informational, clinical and personal support categories [25]. Another qualitative study found that communication about multiple aspects of the care process was a key factor in the patient’s hospital experience with knee and hip replacement [24]. A third qualitative study found both patients and clinicians identified that clinicians needed to provide more consistent clinical attention and improved information to improve care of people with osteoarthritis [47]. Other qualitative and quantitative studies of knee and hip replacement experiences identify the importance of patient education, care continuity, pain management and other physical, psychological and social aspects of the patient experience [48, 49]. Rather than replicating the existing literature’s current approach of listing themes and categories within a single study, future work could use our study’s support framework as an impetus for a deeper understanding of the kinds of support important to patients and the interrelationships between these types of support.

Using a qualitative approach means that our study is limited in its ability to generalize to populations as qualitative research can only be generalized to theory rather than populations [34]. However, having interviewed people from across the province, with purposeful sampling guided by advance knowledge of the variation across our large cohort sample, means that our ability to theorize is rich and varied [50]. Our results contribute relevant insights in understanding the patient experience and the related needs for different types of support for a variety of TKA patients.

This study interviewed participants 8 months post-surgery; different information and perspectives may have arisen had we interviewed people closer to their surgery. However many participants described their TKA experience across time, including shortly after surgery. We only explored patient experience with TKA for those patients who can read English and were well enough to participate in an in-depth interview. Our overall study and the qualitative portion included few persons from racial and ethnic minority groups. We are unable to say whether our results apply to patients more marginalized or challenged in their TKA experience through language or racial or minority status [51, 52]. These results do not explore the experiences of those who never receive TKA surgery in the first place, either by their decision to not have surgery or by being less likely to be offered surgery [53]. Nevertheless, our study’s results provide insight into a wide variety of experiences and the importance of various types of support in TKA patient experience.

## Conclusions

The three domains of support identified in this study—informational support, clinical support, and personal support—can provide guidance for clinicians and other health care system decision makers on areas needing improvement for patient experience and satisfaction with TKA surgery. Clinicians and other decision makers should also keep in mind that patient needs and expectations for support are shaped both before and after TKA surgery. With attention to patient-centred care and a better understanding of the existence and needs of these three domains of support, clinicians and other decision makers can take appropriate actions that could potentially improve the overall patient experience and satisfaction with TKA.

## Abbreviations

TKA: Total knee arthroplasty

## Footnotes

1 Our research team was highly interdisciplinary; as such our analysis for this paper was informed by persons trained in at least one of the following disciplines: health services and policy research, population and public health, health economics, psychology, epidemiology, political science, nursing, and orthopedic surgery.
